# Invalid Self-Assessment of Olfactory Functioning in Parkinson's Disease Patients May Mislead the Neurologist

**DOI:** 10.1155/2020/7548394

**Published:** 2020-11-16

**Authors:** Nele Schmidt, Laura Paschen, Karsten Witt

**Affiliations:** ^1^Department of Neurology, University Oldenburg, Steinweg 13–17, Oldenburg 26122, Germany; ^2^Department of Neurology, University Medical Center Schleswig-Holstein, Arnold-Heller-Street 3, Kiel 24105, Germany; ^3^Research Center of Neurosensory Sciences, University Oldenburg, Steinweg 13–17, Oldenburg 26122, Germany

## Abstract

Olfactory dysfunction (OD) is a prominent nonmotor symptom in Parkinson's disease (PD), and OD is a supportive diagnostic criterion for PD. Physicians often ask their patients if they have noticed a smell disorder. This study evaluates the diagnostic validity of OD self-assessment in PD. To this end, 64 PD patients and 33 age-matched healthy controls were enrolled in a study assessing subjective and objective olfactory functioning. To examine subjective olfactory abilities, first, patients and controls had to classify their olfactory sense as “impaired” or “unimpaired,” comparable to a realistic situation in an outpatient setting. Second, to evaluate subjective olfactory acuity, a visual analogue scale (VAS) was used. Third, the Sniffin' Sticks test battery was used as an objective instrument to diagnose OD. Categorical olfactory self-assessment predicts the classification normosmic versus hyposmic based on the global Sniffin' Sticks score (TDI) with a sensitivity of 0.79 and a specificity of 0.45. TDI correlated significantly with the VAS (*r* = 0.297, *p* = 0.017). The ROC curve analysis, using the VAS rating as a predictor for objective olfaction, revealed 42 as the best possible cutoff score with an area under the curve of 0.63. These results demonstrate that olfactory self-assessments show a low accuracy and are not suitable for the diagnosis of a smell disorder in PD. Objective measures are necessary to evaluate olfactory sense in clinical and research settings.

## 1. Introduction

Olfactory dysfunction (OD) is a common nonmotor symptom in idiopathic Parkinson's disease (PD). As olfactory deficits usually occur in the premotor stage of the disease, they are of particular importance with regard to an early PD diagnosis. OD is a supportive criterion for PD according to the Movement Disorder Society clinical diagnostic criteria for Parkinson's disease [1] and a relevant risk marker for prodromal PD with a positive likelihood ratio of 6.4 [2]. Furthermore, it plays a relevant role in the differential diagnosis of Parkinsonian syndromes [3]. Contradictory to the diagnostic importance of OD in PD, there is no established standard for olfactory assessment in clinical practice. Objective olfactory tests are time-consuming, and therefore, many clinicians simply ask the patients for olfactory impairments and may trust in their subjective evaluation [4]. This also applies to some studies, when subjective olfaction rating was used instead of objective olfactory measures. This approach seems questionable as it could be shown that self-reports of OD do not reflect objective OD in healthy controls [5–9].

Furthermore, a few studies reported subjective unawareness of objective hyposmia or anosmia in PD [10–15]. However, all of these studies used odor identification tests only to assess objective olfactory functioning and some of them reported the relation between subjective and objective olfactory abilities only descriptively as secondary findings. To our knowledge, there is only one published study examining the relation between subjective and objective olfaction in PD which conducted a test battery including several olfactory subfunctions (identification, discrimination, and identification) [16]. The authors reported that 35.9% of the study sample were unaware of an objective smell order. However, it should be noted that the disease duration of the examined PD patients was long (11.1 ± 5.4 years), and almost 40% were smokers which is why the study sample may have to be regarded as not representative in some aspects. Moreover, the patients were only asked one yes/no question regarding the presence of anosmia/hyposmia without further grading. To get more detailed information about the relation between subjective and objective olfaction in PD, our aim was to simulate a realistic examination situation to systematically investigate if olfactory self-assessment is a valid procedure to diagnose OD in PD patients in clinical or research settings. To avoid test selection bias, we used a comprehensive olfactory test battery for assessing subjective and objective olfaction.

## 2. Methods

In a prospective study, we analyzed the data from 64 PD patients diagnosed according to the UK Parkinson's Disease Society Brain Bank criteria [17] and 33 age-matched healthy controls. Exclusion criteria were any neurological disease other than PD, deep brain stimulation, dementia according to the Movement Disorder Society Task Force criteria for dementia associated with PD [18], traumatic or postinfectious damage of the olfactory system, and acute olfactory system disease (e.g., rhinitis and sinusitis). In PD patients, all examinations were performed under regular antiparkinsonian medication. The study was approved by the local ethics committee. All participants provided their informed consent in written form.

Demographic and clinical data of the study sample are given in Table 1. To assess the subjective perception of olfactory functioning, patients and controls were first asked how they evaluate their own olfactory sensibility. They had to choose one of the categorical answers “unimpaired,” “impaired,” or “increased.” Since none of the participants indicated an increased olfactory sensibility, we have not considered this point any further in our analysis. Furthermore, the subjective olfactory abilities were rated using a 14 cm long visual analogue scale (VAS) with “I can smell nothing at all” on the left and “I can smell perfectly” on the right extreme. Scores were indicated as percentages. Afterwards, we conducted the Sniffin' Sticks test battery (Burghart Messtechnik GmbH, Wedel, Germany) as an objective measure for olfactory functioning. In the odor threshold test, the test person has to identify an *n*-butanol smelling felt-tip pen out of three in a total of seven single-staircase trials. The highest concentration is presented initially to become familiar with the scent. Afterwards, the concentration is being successively reduced as soon as the previous one has been correctly identified twice. The odor threshold is defined as the averaged last four correctly identified dilutions (maximum of 16, higher scores indicate better odor threshold). In the odor discrimination test, the participant has to identify one out of three pens which smells different from the other two. The test value is the sum of correct answers (maximum of 16). The odor identification test contains the presentation of everyday odors. The test person has to decide which out of four multiple-choice answers appropriately describes the given smell. The identification score is defined as the sum of correct trials (maximum of 16). Furthermore, the sum of all three subscores (TDI) is used as a global score of olfaction. To classify the study sample in normosmic and hyposmic, we used age- and sex-specific normative data [19]. In the patient group, disease severity was rated with the Unified Parkinson's Disease Rating Scale III and graded according to the Hoehn and Yahr stages. To rule out severe depression as a confounding factor, the 15-item version of the Geriatric Depression Scale was applied.

Statistical analyses were carried out using SPSS 23 (IBM SPSS Statistics for Windows, IBM Corp., Armonk, NY, USA). Given the fact that the Sniffin' Stick test scores were not normally distributed, we used nonparametric statistical tests. To reveal associations between subjective and objective olfactory abilities, Spearman's rank correlation coefficients were calculated. For group comparisons, Mann–Whitney *U* tests or Fisher's exact tests were conducted, respectively. The level of significance was determined at 0.05. Sensitivity, specificity, positive and negative likelihood ratios, positive and negative predictive values, and corresponding confidence intervals (CI) were used to examine the categorical rating of a person's olfactory sense as a classifier for olfactory impairment. To evaluate the diagnostic value of the VAS, a receiver operating characteristics (ROC) curve analysis was performed using SigmaPlot version 11.0 (Systat Software, Inc., San Jose, CA, USA).

## 3. Results

**Figure 1:**
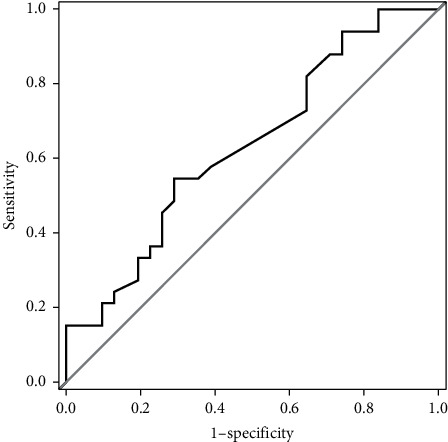
Receiver operating characteristic (ROC) curve of the visual analogue scale (VAS) for PD patients. VAS ranging from 0 to 100% was used to evaluate the subjective olfactory acuity. The ROC curve demonstrates sensitivity (true positive rate) and 1  −  specificity (false positive rate) of the VAS according to an objective diagnosis of hyposmia or normosmia.

All results of the olfactory assessment can be seen in Table 1. Out of 64 PD patients, 31 (48.4%) were objectively classified as normosmic and 33 (51.6%) as hyposmic; 43 patients (67.2%) judged their olfactory sense as being reduced and 21 (32.8%) as unimpaired. In the patient group with subjective OD, 26 patients (60.5%) were objectively classified as hyposmic, while the olfactory functioning of the other 17 patients (39.5%) was rated as unimpaired. Moreover, 7 patients (33.3%) of those without subjective OD showed hyposmia in the Sniffin' Sticks test battery, while 14 patients (66.7%) were objectively classified as unimpaired. The self-assessment of an “unimpaired” or “impaired” olfactory sensibility predicted the actual classification based on the TDI score with a sensitivity of 0.79 (95% CI: 0.63–0.90) and a specificity of 0.45 (95% CI: 0.29–0.63). Positive and negative likelihood ratios were 1.44 (95% CI: 1.0–2.1) and 0.47 (95% CI: 0.22–1.00), respectively. Positive predictive value was 0.6 (95% CI: 0.46–0.74), and negative predictive value was 0.67 (95% CI: 0.45–0.84). Out of all PD patients, 26.6% (*n* = 17) underestimated and 10.9% (*n* = 7) overestimated their olfactory functions. The VAS correlated significantly with the TDI score (*r* = 0.297, *p* = 0.017), odor threshold (*r* = 0.333, *p* = 0.007), and odor identification (*r* = 0.279, *p* = 0.026) in the PD group. When considering only normosmic PD patients, there were no significant correlations between VAS and Sniffin' Sticks scores (all *p* > 0.1*p* > 0.1). In hyposmic PD patients, however, the VAS score correlated significantly with odor threshold (*r* = 0.386, *p* = 0.026), while the correlations with odor discrimination, odor identification, and TDI score did not reach statistical significance. The ROC curve analysis, using the VAS rating as a predictor for the objective olfactory classification, revealed 42 as the best possible cut-off point (according to the maximum sum of sensitivity and specificity) to discriminate normosmic and hyposmic PD patients with a sensitivity of 0.71 and a specificity of 0.55. The area under the curve was 0.63 (Figure 1). In the patient group, there were no significant correlations between Sniffin' Sticks test scores and years of education, UPDRS III, Hoehn and Yahr stages, or Geriatric Depression Scale (all *p* > 0.05).

The healthy control group performed significantly better than the PD group in all olfactory test scores (*p* ≤ 0.001). All 33 healthy controls were normosmic, while 6 of them (18.2%) rated their olfactory sense as reduced. As there were no hyposmic healthy controls, binary classification measures could not be calculated. There were no significant correlations between VAS rating and TDI score (*r* = 0.151, *p* = 0.403) or Sniffin' Sticks subscores (*p* > 0.1). In healthy controls, objective olfaction did not correlate significantly with the education level or Geriatric Depression Scale (all *p* > 0.05).

## 4. Discussion

In the present study, we found low to moderate predictive values of self-reported OD in PD patients. There were significant correlations between a subjective VAS rating of olfactory abilities and an objective olfactory test battery. However, the relationship between the two measures was not strong enough to predict objective olfactory abilities by means of subjective evaluation. The insufficient quality of prediction rate is also expressed by an area under curve that was barely above that of performance by chance. Therefore, the information obtained from the question about subjectively experienced OD is not valid and therefore uninformative.

Our results are in accordance with some other studies that could show a lack of self-awareness regarding OD. While some examinations demonstrated that self-reports do not reflect objective olfactory functioning in healthy controls or nonparkinsonian patient groups [4–9], there are few previously published studies concerning the relationship between subjective and objective olfaction in PD patients. For example, Walter et al. [11] diagnosed hyposmia in 96 out of 115 PD patients according to the Sniffin' Sticks 12-item identification test. Of these patients, 17 (18%) were subjective normosmic. Passali et al. [16] reported that 91.0% of their study sample (*n* = 78) objectively had OD according to TDI results, while only 55.1% reported subjective hyposmia. Kawasaki et al. [13] found significant correlations between odor identification score and a self-administered olfactory questionnaire score (*r* = 0.50, *p* = 0.0043). In a more detailed study of metacognition in PD, White et al. [14] demonstrated an overestimation of olfactory abilities in 63% of their PD group. The authors did not find significant correlations between the University of Pennsylvania Smell Identification Test scores and self-rating (Olfactory Ability Questionnaire) which is probably due to the small study sample (19 PD patients vs. 64 in the present study). In a recently published study conducted by Leonhardt et al. [15], the authors showed that 52% of their PD study sample (*n* = 124) overrated their smell identification ability evaluated on a scale from 0 to 10. In accordance with these studies, we could show that neither a categorical self-assessment nor a self-assessment on a continuous variable is a reliable source of information regarding olfactory impairments in PD. It is noteworthy that in the previous PD studies, a tendency to overestimate olfactory ability was found, while in the present study, the percentage of patients overestimating olfaction was smaller than that of those who were underestimating their olfactory abilities. One aspect to explain this difference could be the different olfactory test measures and normative values used. It should be noted that almost all of the previous studies only used odor identification tests that include a strong memory component affecting the test results [20]. The strength of our study is the application of a full-objective olfactory assessment that included also odor discrimination and odor threshold detection.

Furthermore, in our study, the correlations between subjective and objective olfactory abilities were higher in PD patients than in controls. This is in line with a study conducted by Haxel et al. [21] who showed that the predictive value of subjective smell assessment is higher in smell-impaired patients than in healthy controls. According to the authors, the decisive factor in this context might be that patients with olfactory disorders already focused on their olfactory abilities. This explanation can be applied to PD patients as well because they often suffer from olfactory impairments many years before the diagnosis and is supported by our finding of correlations between subjective and objective olfactory functioning in hyposmic but not in normosmic patients. Although, it should be noted that also in our study sample, the inability to self-assess olfactory performance is not only specific to PD patients but also affects healthy controls.

There are a few limitations to our study. First, the sample size, especially in the healthy control group, was small which is why our results must be confirmed by means of a larger sample in future studies. Second, there were significant differences between PD patients and healthy controls in depression as well as in education level. As we did not find significant correlations between those variables and the Sniffin' Sticks test results in both groups, we do not assume a relevant impact on the study results. Moreover, a recently published study did not find significant differences in the educational level between normosmic and hyposmic/anosmic people indicating that there is no relevant relationship between education and olfactory functioning [22].

In summary, it could be shown that self-reported OD is not a valid procedure to measure olfactory disorders in PD. To prevent the neurologist from being misled, we recommend the use of a standardized and validated olfactory test to diagnose olfactory disorders in PD patients.

## Figures and Tables

**Table 1:** Sociodemographic, clinical, and olfactory characteristics of the study sample.

A: sociodemographic and clinical characteristics				
	PD (*n* = 64)	HC (*n* = 33)	Z	*P* value
Age (years)	64.6 ± 7.5	62.8 ± 6.3	−1.40^a^	0.161
Sex (M/F)	39/25	14/19	3.01 (*χ*^2^)^b^	0.091
Education (years)	13.8 ± 2.7	15.3 ± 2.7	−2.46^a^	0.014
MMSE	28.6 ± 2.0	28.8 ± 1.2	−0.71^a^	0.476
Geriatric Depression Scale	3.3 ± 3.0	1.3 ± 1.1	−3.27^a^	0.001
Apathy Evaluation Scale	11.7 ± 9.4	9.2 ± 7.0	−1.16^a^	0.245
Disease duration (years)	6.3 ± 5.2	—	—	—
UPDRS III	19.0 ± 10.9	—	—	—
L-Dopa equivalent daily dose (mg)	716.9 ± 387.1	—	—	—
Hoehn and Yahr stages	I: 17; II: 24; III: 20; IV: 3	—	—	—
Smoker status (smoker/former smoker/never smoker)	7/22/35	5/9/18	0.64 (*χ*^2^)^b^	0.714
Pack-years	9.1 ± 12.6	6.4 ± 10.6	−0.62^a^	0.534

B: olfactory characteristics				
	PD (*n* = 64)	HC (*n* = 33)	Z	*P* value

Odor threshold test	4.8 ± 3.2	7.4 ± 2.4	−4.16^a^	≤0.001
Odor discrimination test	8.2 ± 2.5	11.8 ± 1.6	−6.24^a^	≤0.001
Odor identification test	7.5 ± 3.3	13.6 ± 1.8	−7.05^a^	≤0.001
TDI	20.5 ± 7.0	32.8 ± 3.9	−6.83^a^	≤0.001
Classification according to Hummel et al. [16] (no. of subjects)	Normosmia: 31, hyposmia: 33	Normosmia: 33, hyposmia: 0	25.79 (*χ*^2^)^b^	≤0.001
Olfactory self-evaluation classification (no. of subjects)	Unimpaired: 21, reduced: 43	Unimpaired: 27, reduced: 6	20.92 (*χ*^2^)^b^	≤0.001
Visual analogue scale	44.2 ± 23.3	61.3 ± 19.0	−3.79^a^	≤0.001

Data are given as mean ± standard deviation. a: Mann–Whitney *U* test and b: Fisher's exact test. PD: Parkinson's disease; HC: healthy control group; MMSE: Mini-Mental Status Examination; UPDRS: Unified Parkinson's Disease Rating Scale; TDI: Global score of olfaction consisting of threshold, discrimination, and identification subscores of the Sniffin' Sticks test battery.

## Data Availability

The data used to support the findings of this study are available from the corresponding author upon request.
